# Health Education in the Curriculum of Early Childhood Education in Galicia, Spain: An Analysis of Decree 150/2022

**DOI:** 10.3390/healthcare13131499

**Published:** 2025-06-23

**Authors:** Ana Gigirey-Vilar, Rubén Navarro-Patón, Santiago Martínez-Isasi, José Eugenio Rodríguez-Fernández

**Affiliations:** 1 Facultad de Ciencias de la Educación, Universidade de Santiago de Compostela, 15782 Santiago de Compostela, Spain; ana.gigirey.vilar@gmail.com; 2 Facultad de Formación del Profesorado. Universidade de Santiago de Compostela, 15782 Santiago de Compostela, Spain; ruben.navarro.paton@usc.es; 3 Facultad de Enfermería, Universidade de Santiago de Compostela, 15782 Santiago de Compostela, Spain; santiago.martinez.isasi@usc.es

**Keywords:** health education, early childhood education, physical activity, educational curriculum, early childhood school

## Abstract

**Introduction:** The entry into force of a new education law (LOMLOE) in Spain introduces a regulatory framework that is committed to Health Education (HE) in the school context. Schools are considered strategic settings for acquiring healthy patterns that can last a lifetime, as confirmed by major European organizations such as the World Health Organization (WHO) and the European Commission (EC). The objective of this study was to conduct an expert consensus analysis of Decree 150/2022, on Early Childhood Education (ECE) curriculum in the region of Galicia (Spain), with regard to aspects related to HE. The analysis focused on issues related to physical, mental/emotional and social health and included comparisons with curricula from other regions of Spain and other European countries. **Method:** To this end, a panel of four experts (from the fields of nursing and physical education) was assembled to conduct a consensus analysis of the legislative document, using a mixed-method approach that combined the Delphi technique with structured face-to-face consensus meetings. **Results:** The summary of the final proposal reflects a document aligned with societal needs regarding children’s health while also addressing mental/emotional and social health. **Conclusion:** The proposal aims to promote the acquisition of healthy lifestyle habits that are sustained over time. However, the success of the document in terms of both content and practical application remains uncertain. Further studies will be needed in the coming years to analyze and provide concrete evidence regarding its outcomes and impact on students.

## 1. Introduction

The World Health Organization [1] refers to Health Education (HE) as a set of pedagogical actions and opportunities to communicate and improve health literacy, including enhancing health knowledge and developing skills that promote individual and collective well-being. But this definition is incomplete if its orientation is focused on the medical field; it is necessary to think of it as a training that seeks well-being in its entirety [2], which is why in recent years educational centers have been considered a central pillar in this type of training and the promotion of healthy habits.

Various authors [3], in a complex analysis of what HE encompasses, have referred, in general, to physical, mental/emotional and social health and, specifically, to key words such as sedentary lifestyle, overweight, obesity, eating habits, hydration habits, sleeping habits, rest and routines, physical activity and sport, healthy spaces, improper use of new technologies (especially screens), prevention of violence or prevention and action in the event of accidents and/or injuries. Aspects such as these, as well as whether educational centers are considered strategic places for their knowledge, treatment and development, should be included in educational laws and decrees at the different stages of the education system.

The entry into force of a new education law [4] and the recent decree on Early Childhood Education (ECE) in the Autonomous Community of Galicia, Spain [5], show the intention for a regulatory framework committed to ECE in the school context and the awareness of the importance of childhood and the acquisition of patterns that can last a lifetime. In addition to key aspects, such as those listed by authors specializing in educational curriculum [3], there are other specific aspects, such as healthy eating [6], emotional management [7] or accident prevention, transmitted through dynamic, interactive and child-centered methodologies [8] and in collaboration with different educational agents [9].

In Spain, as a result of Organic Law 3/2020 [4], a new education law was created with the aim of adapting the education system to the challenges of the 21st century and in accordance with the objectives set by the European Union and UNESCO for the decade 2020–2030. The Royal Decree—RD 95/2022 [10]—was developed with the aim of fixing and establishing the minimum teaching of ECE throughout the Spanish territory, proposing an overall vision for the whole country. Based on this Royal Decree, each Spanish Autonomous Community will specify in a document specific regulations adapted to its characteristics and peculiarities as a region (language, customs, etc.) but always maintaining the basis of this minimum decree, which unifies criteria and procedures for the whole state. For this reason, the various educational curricula for ECE in Spain are very similar, except for the particular specifications for each region, as mentioned above.

In Europe, the promotion and inclusion of EI in school curricula is also reflected in different instruments and documents. The European Commission [11] highlights that interventions in ECE can have long-lasting effects on the adoption of healthy behaviors. The same body states that in ECE, children should acquire knowledge and skills related to hygiene [12], healthy eating [13], physical activity [11] and risk and accident prevention [14].

The main European countries also recognize the importance of integrating HE content in ECE. Although approaches and terminologies may differ, they all agree on the need to promote healthy habits, hygiene and emotional well-being from an early age, thus laying the foundations for healthy and balanced development. In England, the Early Foundation Stage includes competences related to health and well-being, promoting the teaching of healthy habits, personal hygiene and the importance of physical exercise from an early age in their daily activities [15,16]. In Germany, the curriculum framework for ECE (Bildung für Kinder) incorporates content on physical and emotional health, promoting healthy eating, hygiene, physical activity, emotional awareness and self-care [16,17]. And, in France, the curriculum for école maternelle (ECE) includes a health and hygiene promotion program, focusing on physical exercise, balanced nutrition, personal hygiene habits and emotional well-being [16,18].

The need to carry out promotion and HE interventions in educational centers has been recommended by various national and international bodies (WHO, UNESCO, Council of Europe, Spanish Ministry of Education, etc.). As early as the European Conference on EfS in Dublin in 1990, the inclusion of HE in educational curriculum was recommended as the most effective way to promote healthy lifestyles over the long term, especially among young people, regardless of their social class, gender and family economic level [19].

HE in the field of ECE is a fundamental tool for creating healthy habits from the earliest stages of life. The curriculum itself states that it is at this stage that intentions should be created and gradually developed in depth at later stages. In the same way that this can happen with language acquisition during the earliest years (which strengthens adherence to language in successive years), healthy habits are in the same situation: conditions are created during early ages for development and consolidation in successive years. HE in ECE not only positively influences children’s well-being but also contributes to the construction of a more aware and responsible society in terms of public health [20].

Basic concepts related to one’s own body, such as hygiene, nutrition, rest (sleep), physical, psychological, emotional and affective-sexual and social, as well as actions to prevent age-specific risks and injuries or interventions to modify the environment to promote sustainability, constitute the basis of the HE that should be established in ECE, using methodological strategies and didactic resources appropriate to the age, context and educational intentions [21].

However, one of the most important aspects to be promoted at this stage is physical activity and reduction in sedentary lifestyles, with a commitment to more time for motor engagement and moments of activity during the school day (motor skills sessions, active breaks, interdisciplinary projects, methodologies that emphasize learning through movement, etc.), as well as activity before and after the school period (active travel, extracurricular activities, etc.) [22]. Let us not forget that the problems of physical inactivity and sedentary lifestyles are some of the most important public health problems worldwide, with extremely alarming data from Spain [23].

The aim of this research was to carry out an expert consensus analysis of Decree 150/2022, which establishes the organization and curriculum of ECE in the autonomous community of Galicia (Spain), and its aspects related to HE, assessing the issues related to physical, cognitive, affective/relational and social health and making comparisons with other regions of Spain and different European countries.

## 2. Materials and Methods

In order to ensure an interdisciplinary approach, profiles from different fields were integrated, all with relevant knowledge of the content analyzed. For the content analysis and validation process, a panel of four experts (two in the field of nursing and two in the field of physical education) with specialized training and proven experience in the relevant fields of study was formed. The selection criteria included the following:

(a) Second- or third-cycle university degree in areas related to the object of analysis;
(b) At least five years’ experience in teaching, research or professional development in the specific area;
(c) Academic production (publications, papers or technical reports) linked to the subject under study.

**Figure 1:**
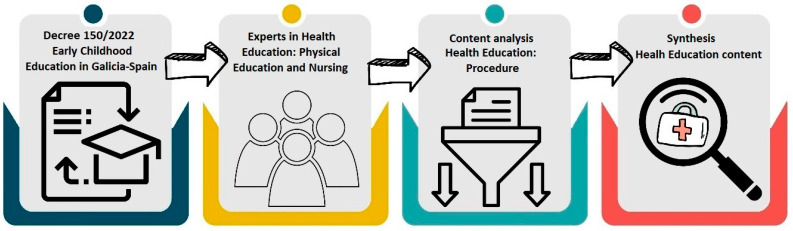
Extraction process and content analysis of Decree 150/2022.

The methodology consisted of a consensus analysis approach [24,25] on the contents of HE within the official ECE curriculum in the autonomous community of Galicia, Spain [5]. This methodology corresponds to a mixed-methods approach combining the Delphi procedure and structured face-to-face consensus [24], developed in such a way that it contains the key elements that integrate and replicate this methodology (selection of experts, definition of objectives and criteria, individual assessment, rounds of group discussion, final consensus and report writing). It is, therefore, a methodology similar to a modified Delphi method, with structured double-round voting that integrates the logic of the consensual analysis of a panel of experts. This group of experts analyzed all contents of the Decree, shaping and synthesizing the final proposal, as reflected in the results of this study (Figure 1).

The purpose of the analysis was to identify and thematically categorize HE-related content in the ECE curriculum, following a qualitative approach based on previously defined criteria [26], which included the following:

(a) Clarity or degree of understanding of the content;
(b) Coherence or internal consistency;
(c) Relevance of the content in relation to the research objectives;
(d) Sufficiency or items necessary for its complete evaluation;
(e) Relevance or appropriateness to the educational context and to the training stage (ECE).

The resulting categorical system included references to the following main categories, subcategories and indicators (Table 1).

The evaluation and analysis of the legislative document by this group of experts was carried out with the aim of guaranteeing the validity, coherence and quality of the information presented in the results (Section 3) of the study. Therefore, a consensus opinion is offered by the group, integrating different perspectives, reducing individual biases and providing a final global agreement that gives strength and credibility to the analysis carried out—a final agreement that translates into a majority opinion or qualitative consensus.

The procedure followed for the analysis of the legislative document was as follows:

- Selection of the group of experts. Due to the content to be dealt with, a multidisciplinary team with relevant knowledge on the subject matter of the document was considered. Thus, 2 experts in the area of nursing and 2 experts in PE, all with higher degrees and proven experience in their field, took part in the analysis;
- Definition of objectives and criteria. With the aim of identifying the content related to EoS in the pre-school education curriculum, the criteria to be selected were established based on the WHO’s guidelines [27] on school HE, specifically, content related to physical, social and mental health—generally, content related to healthy habits and lifestyles;
- Individual evaluation phase. Each expert carried out an independent evaluation of the document, applying the previously defined criteria;
- Group discussion phase (first round). In a face-to-face meeting, the individual evaluations of each expert were discussed and shared. Preliminary proposals were elaborated and points of agreement and disagreement were identified;
- Individual analysis phase of the preliminary proposals. Each expert individually analyzed the proposals derived from the first group-discussion phase, further analyzing the points of disagreement in an attempt to come closer to a general consensus;
- Group discussion phase (second round). This was carried out to resolve differences, clarify positions and seek common agreement;
- Formation of the consensus and drafting of the final report. Always by agreement and voting system, an agreement is reached on the final content to be included in the analysis (see Section 3).

## 3. Results

After analyzing Decree 150/2022, which establishes the organization and curriculum of ECE in the Autonomous Community of Galicia, Spain, in relation to HE, the document contains the following information in the preamble, titles and annexes:

### 3.1. Preamble and Titles

Organic Law 2/2006, of 3 May, on education, recently amended by Organic Law 3/2020, of 29 December, establishes that the curriculum must be aimed at facilitating the educational development of students, guaranteeing their comprehensive education, contributing to the full development of their personality and preparing them for the full exercise of human rights and active and democratic citizenship in today’s society, without, in any case, being a barrier that generates school dropouts or prevents access to and exercise of the right to education (p. 47984).

In the document, mention is made of a series of changes aimed at both the renewal of teaching practice and the teaching–learning process, establishing new approaches to learning and assessment, aspects which will entail a significant change in the tasks for students and innovative didactic proposals (p. 47986).

#### 3.1.1. Preamble

In relation to HE, this part of the document states that Title III is dedicated to the different relevant and strategic educational plans for the Galician education system, linked to the needs of a system aimed at providing students with the crucial competences for citizenship in the 21st century. It is at this point that reference is made to the fact that, in addition to the school library plan and the digital education process, ‘emphasis is placed on the promotion of healthy lifestyles among pupils at this stage, which is essential for their development’ (p. 47987).

#### 3.1.2. Preliminary Title (General Provisions)

In this part of the curriculum, it is important to highlight the aims of IE, which, although not explicitly referring to HE, can be extracted from the purpose of the stage, which is ‘to contribute to the comprehensive and harmonious development of students in all its dimensions, as follows: physical, emotional, sexual, affective, social, cognitive and artistic, fostering personal autonomy and the progressive creation of a positive and balanced self-image, as well as education in civic values for coexistence’ (p. 47989). Likewise, in the stage objectives, it is possible to generalize proposals oriented toward HE in the physical sphere (‘To know their own body and that of other people, as well as their possibilities of action…’ or ‘To progressively acquire autonomy in their habitual activities’), psychic/emotional (‘To develop their emotional and affective capacities’) and social (‘To relate to other people in equality and to progressively acquire elementary guidelines for coexistence and social relations…’) (pp. 47991–47992).

#### 3.1.3. Title I (Development of the Curriculum)

- Article 15. Pedagogical principles. In the two cycles of this stage, attention will be paid progressively to affective development, emotional management, movement and body control habits… to patterns of coexistence and social relations (p. 47998). Specifically, in point 8, it is specified that ‘education for responsible and sustainable consumption, and the HE promotion that encourages healthy habits shall be included’ (p. 47998). Point 9 adds that girls and boys should be encouraged to acquire personal autonomy and to develop a positive, balanced, egalitarian self-image free of discriminatory stereotypes (p. 47998).
- Article 16. Transversal elements. 1. In ECE, at least those contents of a cross-curricular nature that are included in numbers 7, 8 and 9 of article 15 shall be dealt with (p. 47998). As mentioned above, HE and the promotion of healthy habits are among these contents.

#### 3.1.4. Title III (Educational Plans)

- Article 31. Promotion of healthy lifestyles (p. 48006). It is explicitly stated that ‘Schools must include within their educational and functional project a plan of physical activities and healthy habits with the aim of daily practice of sport and physical exercise during the school day and the promotion of an active, healthy and autonomous life, on the part of pupils, which will be specified annually in the annual general program through the corresponding actions’ (p. 48006).

### 3.2. Annex I. Key Competences

ECE is the beginning of the process of developing the skills and abilities necessary for the acquisition of the key competences for lifelong learning that appear in the Recommendation of the Council of the European Union of 22 May 2018. These competences are Competence in Linguistic Communication (CLC), Multilingual Competence (MC), Mathematical Competence and Competence in Science, Technology and Engineering (MCCSTE), Digital Competence (DC), Personal, Social and Learning to Learn Competence (PSLLC), Citizenship Competence (CC), Entrepreneurial Competence (EC) and Competence in Cultural Awareness and Expression (CCAE).

The acquisition of these competences throughout their schooling is expected to prepare students to successfully face the main challenges of the 21st century, as follows: planning healthy lifestyles, protecting the environment, resolving conflicts peacefully, acting as responsible consumers, using technology ethically and effectively, promoting gender equality, managing the anxiety generated by uncertainty, identifying situations of inequity and developing feelings of empathy and cooperating and living together in open and changing societies, among others (pp. 48008–48009).

In the first cycle, special relevance is given to the processes of knowledge and mastery of one’s own body and individuation, to the construction of a network of relationships and interactions in physical and social environments and to the use of languages that make this possible. All of this is governed by the fundamental principle of respect for the individual rhythms of each girl and boy, for their essential care in an affective, participative and equal environment that provides them with confidence, well-being and security (p. 48009).

In the second cycle, previously acquired learning is expanded and reinforced, and the acquisition of skills that contribute to “learning to be” and “learning to do” is intensified in order to advance toward the development of a certain degree of autonomy, responsibility and initiative in the performance of tasks (pp. 48009–48010). Explicit references to the acquisition of healthy habits or to HE in general can only be found in the following three of the eight key competences: MCCSTE, DC and CC (Table 2).

### 3.3. Annex II. Curriculum for the Area of “Communication and Representation of Reality”

The area of Communication and Representation of Reality aims to promote in children the skills that enable them to communicate and interpret reality through different languages and forms of expression as a means of constructing their identity, representing reality and relating to other people. The objectives of the area relate to the ability to communicate effectively with others in a respectful, ethical, appropriate and creative manner (p. 48013).

Children are immersed in a society in which digital technology affects the way we communicate, obtain information, learn and relate to each other. It is, therefore, the teacher’s responsibility to establish guidelines for the development of healthy habits for the use of digital tools and technologies, mainly referring to the times and moments of use by pupils, the differentiation between image and reality and access to appropriate content. A digital literacy process should, thus, be initiated from the earliest educational stages, focusing on students’ understanding of the creative and communicative roles of technological tools, beyond passive leisure use and content consumption (p. 48015).

In relation to the objectives, none of the five listed in this area refer explicitly to HE. The same applies to the pedagogical guidelines, where there are no references to HE. Table 3 shows the assessment criteria and contents for the first and second cycles.

### 3.4. Annex II. Curriculum of the “Growth in Harmony” Area

The area of Growth in Harmony focuses on the personal and social dimensions of the child, understood as inseparable and complementary, which are developed and regulated in a progressive, joint and harmonious manner, although it only acquires meaning from its complementarity with the other two, as it takes place in a specific physical and natural environment and requires the use of different languages and representations of reality (p. 48031).

Therefore, attention is paid to psychomotor development, the gradual acquisition of self-control and the gradual process of constructing one’s own identity, as a result of the discovery of one’s motor possibilities and interactions with oneself, the environment and others. In this process, the starting point will be the free experimentation with their own motor skills. Progress will be made from total dependence on the adult toward progressive autonomy, insofar as each individual must integrate and use the resources and strategies that facilitate adjusted and adapted development of his or her motor skills (pp. 48031–48032).

The objectives of the area identify the actions that children are expected to be able to carry out in relation to their own personal and social development throughout the stage, as a result of educational intervention. Of the four objectives proposed for this area, the last objective deals with the necessary correlation between the construction of one’s own identity and the interactions in the socio-cultural environment in which it takes place, highlighting the importance of fostering and favoring healthy, sustainable, effective, egalitarian and respectful interactions (p. 48032).

In the early stages of development, the body itself is a source of experimentation, learning, relationships and expression and the basis for autonomous activity. The school environment must provide the appropriate context and the necessary accompaniment under an attentive, patient and respectful eye, so that children can discover the pleasure of self-initiated activity, which is their main need in relation to their environment, in a stimulating atmosphere of security, calm and tranquility. In this way, they will recognize their body, globally and partially, its perceptive and action possibilities and expression and movement, as well as its limitations, and they will be able to identify the sensations they experience, to enjoy them and to use the expressive possibilities of the body to express them (p. 48032).

The needs must be met in a welcoming and calm atmosphere that provides the necessary time for each moment to be experienced as pleasant. It is only from this sense of well-being that the rest of the principles attain significant and global value. Within this framework, school life is organized around stable routines, planned on the basis of biological rhythms and linked to the progressive acquisition of healthy eating, hygiene and rest habits. Gradually, their initiative will be increased to incorporate into their daily practices habits that contribute to the care of their own bodies and the spaces in which they spend their daily lives, in parallel with the development of personal autonomy and the awakening of awareness of the relationship of interdependence and eco-dependence between people and the environment. The individual characteristics of all pupils will be considered when facing and carrying out these routines, for which the necessary times or adaptations will be respected. In this way, progress will be made from complete dependence toward a certain degree of autonomy in the satisfaction of their needs and in the acquisition of sustainable and eco-socially responsible habits (pp. 48033–48034).

In relation to the objectives of this area, objective 3 includes explicit references to HE, as follows: OBJ 3. Adopt models, rules and habits, developing confidence in their possibilities and feelings of achievement to promote a healthy and eco-socially responsible lifestyle.

- The acquisition of healthy and sustainable habits and their progressive integration into daily life contribute to the care of one’s own body, as well as to the achievement of increasing autonomy. In this process, it is essential that they know and reflect on the rules that contribute to creating trends of respectful action with themselves, with others and with the environment, from an interdependent and eco-responsible perspective. It is also expected that they will begin to reflect on the responsible consumption of goods and resources, as well as promoting physical activity as healthy behavior.
- This is transferred to the classroom through the implementation of routines understood as sequenced practices that are repeated in a stable and intentional manner to favor the regulation of biological rhythms and the adjustment to personal time. It is necessary to find moments of personal attention, through individualized treatment of each child, especially in terms of satisfying their needs, respecting their biological rhythms and ensuring their comfort and well-being. All this contributes to the development of a more adjusted perception of oneself and the feeling of achievement derived from the perception of the progressive competence acquired in activities related to food, hygiene, clothing, rest and activity.
- Finally, initiatives related to the importance of risk prevention and accident avoidance should be encouraged, developing strategies that allow students to analyze potentially dangerous situations and act coherently.

Table 4 and Table 5 show the assessment criteria and content for the first and second cycles, respectively.

Regarding the pedagogical guidelines for this area, the lines of action in the teaching–learning process are based on the following:

- The construction of new secure attachments as a starting point for creating sound self-esteem, etc. (p. 48043);
- The promotion of relationships among the people with whom children interact, the positive expression of their self-image and the self-confidence they have for the construction of their own identity (p. 48043);
- Participation in games and psychomotor activities that foster interactions, promoting the development of empathy and resilience, which will lead to a constructive acceptance of both achievements and mistakes and to an appreciation of effort, perseverance and initiative, thus developing all of the children’s potential (p. 48043);
- Leveraging routines and other daily activities as meaningful “moments” that foster globalized work, incorporating the verbalization of actions to internalize their sequences and initiate and consolidate healthy habits and body self-care (p. 48044);
- Planning learning situations that encourage responsible and sustainable consumption, as well as health promotion (p. 48044);
- Creating safe and emotionally positive contexts for children to gain confidence in their capabilities and limitations as a basis for expressing their interests, needs and emotions. This will contribute to their emotional development and the acquisition of frustration tolerance, adapting their behavior to the different situations proposed (p. 48044).

### 3.5. Appendix II. Curriculum for the Area of “Discovery and Exploration of the Environment”

The Environmental Discovery and Exploration area refers to the discovery, exploration and understanding of what shapes children’s reality, considering their multiple relationships and interdependencies, with the goal of building an increasingly integrated understanding of the physical, natural and social environment. The environment is an integrated whole, where natural and cultural elements interact and influence each other. It is important that during this educational stage, children, in addition to identifying the different elements that make up the environment, gradually discover and understand the relationships between different objects, phenomena and events (p. 48045).

This area aims to foster the process of discovery, understanding, observation and exploration of the physical, natural and social elements of the environment, conceived as an element that provokes emotions and surprises. The goal is for children, along with their progressive understanding, to adopt and develop attitudes of respect and appreciation for the need to care for and protect it, thus perceiving the environment as an integrated whole (p. 48045).

In this area, no evidence is collected regarding objectives, content, criteria or other aspects directly related to HE.

## 4. Discussion

This study analyzes Decree 150/2022 [5], under Organic Law 3/2020 [4], in relation to HE in ECE. This review highlights the main content established by the curriculum as actions for HE with children. Briefly, these can be summarized as follows:

- Practicing a healthy lifestyle and promoting comprehensive well-being through a balanced and enjoyable diet, sufficient independent rest, a love of cleanliness and appropriate attire (hygiene);
- Healthy attachment relationships (affective-social sphere);
- Cleanliness, care and order in the environment (sustainable and eco-socially responsible habits);
- Accident and illness prevention;
- Responsible and healthy use of digital technologies.

The continuous references to all these contents fit perfectly with many of the studies currently published on this subject [3,6,7,8], especially given the specificity and timeliness of the contents and work and with the guide edited by the Ministry of Health [21], which is aimed at the entire educational community to facilitate the transformation of centers into Health-Promoting Schools, thereby contributing to the acquisition, development and adherence to healthy habits in childhood, with continuity in later stages and, ultimately, throughout life.

However, there are obstacles that hinder the development of the standard and, consequently, reduce the expected effectiveness of what is specified in the educational curriculum on HE. Firstly, there is a lack of training and awareness among educational staff. Various studies have reported that teachers’ training in the field of PE in the ECE stage has shortcomings, one of which is a lack of specialization [28], an aspect that has a direct impact, leading to insecurity and lack of confidence in teaching this type of content in ECE [29]. In this regard, it should be remembered that studies leading to a degree in ECE for teachers at the University of Santiago de Compostela [30], one of the three Galician universities, only includes one compulsory subject in its syllabus of only 6 ECTS credits and one optional subject of 4.5 ECTS credits, providing a very poor background if the aim is to provide qualified teachers with knowledge on the application of PE in the ECE stage.

In addition to the lack of specialization, several studies highlight the need for ECE teachers to improve their in-service training and participate in specific programs to strengthen their competences in PE in ECE. Although initial training is very important, constant updating would allow teachers to incorporate innovative methodologies, adapt to the changing needs of pupils and enhance their pedagogical competence; it would also help to reinforce teachers’ confidence and motivation, which translates into more effective and engaging teaching [28,29,31,32,33].

Authors such as Martínez and Ruíz [34] carried out a comparative analysis of teacher preparation in PE for ECE in different European countries, highlighting the variability from one country to another, noting the European countries that offer specialized programs and others that integrate training into a more general framework, such as Spain. What they do agree on is that quality and depth of preparation directly influence the competence and confidence of teachers in the teaching–learning process, increasing the quality of teaching. These aspects are also contrasted in other relevant research in this field of study [35,36,37].

Secondly, we must discuss the lack of time provided to address all of these contents effectively. A study by teachers at the University of A Coruña (Spain) [38] found that 16.98% of schools in the ECE stage in Galicia (where this study of educational legislation is framed) did not dedicate any hours per week to physical activities (specific sessions of children’s PE). This fact reflects the seriousness of the issue in the following two ways:

- These 16.98% of ECE schools will not be able to achieve the objectives set out in the educational curriculum, which is compulsory for all teachers and must be reflected in their teaching programs;
- The ECE curriculum in Spain does not specify PE as compulsory. There is no specific subject on PE, but the educational curriculum is developed in a global and holistic way. In the next stage (6–12 years), PE is compulsory for all grades of primary school at 2 h per week. Therefore, the teachers included in this 16.98% are not breaking the rule related to holding specific PE sessions, but, on the contrary, they should explain how they are managing to ensure that their pupils are acquiring the competences and objectives set if not holding these types of sessions.

The lack of time in ECE schools is a particular handicap in achieving the objectives related to the promotion and development of HE. In Spain, various studies [39,40,41,42] reflect the need to increase the number of hours of physical activity in ECE and the impact that this greater amount of time would have on children’s health, as there is no other way of achieving the proposed objectives without the consequent dedication of weekly time.

In Europe, recommendations from important institutions [43,44] related to increasing physical activity in ECE suggest targets should focus on proposing at least 180 min (3 h) of activity per day for pre-school children, including at least 60 min of moderate to vigorous intensity, an approach that goes beyond specific PE sessions, encouraging turning everyday into an active day in which active and outdoor play form an integral part of the daily routine in ECE settings.

Müller and Schmidt [45] point out that, in many European countries, the hours devoted to physical activity in schools are insufficient due to factors associated with the lack of adequate infrastructure, as well as an overload of other academic contents and educational policies that prioritize other areas. In Sweden, Finland and Germany, to give a few examples, initiatives have been proposed to promote movement inside the classroom and in outdoor spaces, and educational policies have been established that effectively integrate physical activity into the educational curriculum, with excellent results [45]. In the same vein, another study [37] shows positive results in pre-school settings in Scandinavia, which are compared on the basis of the different approaches implemented in Sweden, Norway and Denmark, where the promotion of physical activity in early childhood is a priority to foster healthy development.

Despite the obstacles listed above, they can, at the same time, become facilitators that can enhance the success of the implementation of the standard and, consequently, ensure that HE enters schools to its full potential. Aspects such as increasing the amount of time devoted to physical activity, improving teacher training or implementing policies aimed in this direction—always working in a safe and stimulating environment, with the help and participation of families and the use of new and motivating teaching–learning methodologies—are key. In this study, we can see that in Spain there is a lot of content related to HE in ECE, but, nevertheless, PE is not a compulsory subject and initial teacher training is clearly insufficient despite the recent approval of a new Education Law [4].

The issues of physical inactivity and sedentary lifestyle are problems that affect the entire world’s population, and Spain ranks among the highest European countries for childhood overweight and obesity [46]. The University of Zaragoza (Spain) [47] goes further, stating that Spain tops the European ranking for childhood obesity. Studies like these and many others by scientists and organizations dedicated to health and safety around the world, such as the World Health Organization, warn of a problem that must be addressed from the earliest years of life (healthy habits) and from a place that, curiously, encompasses the entire child population (schools). The new EI educational curriculum in Galicia and Spain moves in this direction, but it is not just a matter of a legal text or a properly studied and planned teaching guide; teachers’ training, involvement and commitment must also move in the same direction. Otherwise, the actions established will not have the desired effect [48].

In the review of the document, we find references to issues related to responsible consumption and sustainable development. Aspects such as “responsible and sustainable consumption”, “sustainability practices, environmental care and protection” and “healthy, sustainable, and eco-socially responsible habits” are some of the references mentioned in 150/2022 [5]. This approach to EPS in EI is aligned with the 2030 Sustainable Development Goals (SDGs), specifically the following:

- SDG3 (Good Health and Well-Being): promoting healthy habits during childhood;
- SDG4 (Quality Education): fostering critical thinking and sustainability.

These types of comprehensive actions reinforce awareness of the importance of sustainable development [49], all of which are aspects recognized by the United Nations [50].

The analysis of educational legislation related to HE in ECE reveals important contrasts and convergences with respect to the recommendations of international organizations and comparative policies in different countries. In general, the regulatory frameworks of multiple education systems recognize the importance of incorporating HE from the earliest years of school life; however, they differ significantly in terms of the clarity, systematization and curricular operationalization of such content.

According to the WHO [51], effective HE must be comprehensive, cross-cutting and continuous, addressing not only physical health but also mental/emotional and social health—aspects that are included as the main categories of this study. In this line of Health-Promoting Schools, the WHO [51] and UNESCO [52], in the recent revision of the standards for healthy schools (2021–2023), establish that the contents of HE should be articulated to the educational environment, interpersonal relationships and school community as a whole. However, the physical sphere is still prioritized over the mental/emotional or social sphere [53,54]. In the specific case of Spain, with the recent approval of the new Education Law [4], the decree on minimum education [10] and decree 150/2022 [5], the treatment of HE in the three spheres has been balanced, although the physical sphere still prevails over the others, which coincides with studies on other European countries [53].

Recent research in Europe [55,56] concludes that the most effective school interventions are those that integrate multiple dimensions of health, such as the one currently included in Spain, but other studies [57] warn that when emotional health is secondary and poorly defined in the educational curriculum, there is the risk of problems in HE’s applicability. This was more present in Spanish legislation before the entry into force of the new law, and there are years ahead to check the changes after this legislative transition.

In summary, Decree 150/2022 [5] reflects an intention to align with the international HE agenda, but a gap persists between regulation and practice due, above all, to the aforementioned aspects of deficiencies in teacher training, resources and available time, as well as curricular integration.

Finally, in relation to the educational implications based on the results, the findings underline the urgency of integrating content that address mental and emotional health and well-being into teacher training programs, as well as introducing specific areas of HE in educational programming [54,58]; redistributing time and space during the school day to comply with international WHO recommendations [56]; implementing a comprehensive eco-social approach, linking health, environment and citizenship, thus reinforcing SDGs 3 and 4; conducting institutional evaluation and monitoring to ensure that HE is more than just good intentions; and strengthening inter-sectoral collaboration (families and collaboration of teachers in various areas or fields of knowledge, such as education, physical education and nursing), highlighting the need for synergies between the education, health and social sectors [59].

## 5. Conclusions

In ECE, the curriculum outlines the intentions for the work and development of the HE. Although with a lower content load than in the primary education stage, it considers the basic issues that should be promoted from the earliest years of life. Healthy lifestyle habits related to nutrition, physical activity, personal hygiene and grooming, rest, cleanliness of space, relaxation, sleep and wake rhythms, as well as healthy attachment in relationships, constitute an essential area of intervention at a stage in which lifelong patterns begin to be acquired.

However, the reflection of HE content in the educational curriculum does not always imply its correct application in ECE classrooms. The design of the regulatory framework is adequate, but its implementation is uncertain in terms of its real applicability. The fact that the initial training of ECE teachers is very basic (the curricula confirm this), the lack of continuous training in specific physical activity and PE programs in ECE, as well as the non-obligatory nature of the practice of physical activity and sport at this educational stage, constitute difficulties that must be addressed in an unavoidable way by the competent educational authorities, both at national and international levels.

In fact, given the recent entry into force of the new educational law and the regional legislative decree (year: 2022), it is still risky to establish the success of the document in terms of its content and consequent applicability. The next few years are key to evaluating the document and gathering concrete evidence on the outcomes and its influence on pupils; but, in principle, the inclusion of this type of HPS content and the approach related to the physical, mental/emotional and social spheres constitute a good starting point for HPS work in schools, as recommended by the main international organizations.

The public health problem promulgated by the main European and world bodies should serve as an example so that public policies have an impact on improving the health of all citizens, a chain that must begin at the earliest stages of education.

## 6. Limitations of the Study

The main limitation of this study is the fact that subjective opinions may be established in the expert consensus; however, the procedure established is aimed at reducing this circumstance as much as possible, including in the selection of the experts who evaluated the legislative document. Likewise, the lack of an empirical validation of the study makes it difficult to establish the success of the document’s application, as it is a documentary review and not a research study tested in real scenarios, limiting the production of reliable, applicable results that are coherent with the educational reality studied. Another limitation is that this is a regional document [5], but since its basis is in the minimum decree [10], this means that much of its content can be extrapolated to the whole of Spain.

## 7. Prospects

The findings of this study, as well as the references to the difficulties encountered in the application of HE content in ECE based on the increase in the number of hours dedicated to movement in the ECE classroom, the initial training of ECE teachers, the need for continuous teacher training and the use of new teaching–learning methodologies, should serve to establish effective educational policies in the form of laws and educational decrees that effectively address HE content. In this sense, general guidelines promoted by the main European organizations should establish common content in different countries in order to guarantee minimum teaching standards to be met by all, thus ensuring teaching based on quality and the educational needs of the youngest schoolchildren.

## Figures and Tables

**Table 1:** Main categories, subcategories and indicators.

Main Category	Subcategories	Indicators
Physical health	Physical activity	Incorporation of motor games, psychomotor skills, free movement Recognition of exercise as part of well-being Time dedicated to outdoor movement
	Healthy eating	Inclusion of contents on different foods Promotion of eating habits Practical activities (workshops, conferences, etc.)
	Body hygiene	Body grooming Cleanliness of the immediate environment Hygienic habits in general
Mental and emotional health	Identification and emotional management	Relationship to basic emotions Teaching of strategies to manage emotions Use of stories, games and other emotional resources
	Autonomy and self-esteem	Promotion of decision making Positive reinforcement of effort or achievement Recognition of one’s own and others’ skills
	Digital education	Responsible use of technological tools Digital literacy
Social health and interpersonal relationships	Coexistence and respect	Promotion and use of rules of courtesy and conflict resolution Recognition of prosocial behavior Use of materials that promote diversity and respect
	Risk prevention and safety	Accident prevention Correct use of objects Safety elements and dangers in the immediate environment

**Table 2:** Health Education in the key competences in Early Childhood Education.

Mathematical Competence and Competence in Science, Technology and Engineering (MCCSTE)
Children are introduced to logical–mathematical skills and take their first steps toward scientific thinking through play, manipulation and simple experiments. They are also invited to understand and explain some phenomena of the surrounding natural environment and to learn to appreciate the environment and to acquire healthy habits (pp. 48011–48012).
Digital Competence (DC)
At this stage, the process of digital literacy begins, which involves, among other things, access to information, communication and the creation of content through digital media, as well as the healthy and responsible use of digital tools (p. 48012).
Citizenship Competence CC)
An active commitment to the values and practices of sustainability and the care and protection of animals is encouraged. To this end, the acquisition of healthy and sustainable habits is promoted through routines that the children will integrate into their daily practices. In addition, they establish the necessary conditions to create respectful behaviors with themselves, with others and with the environment, which prevent discriminatory behavior of any kind (pp. 48012–48013).

**Table 3:** Evaluation criteria and contents of Communication and Representation Area of Reality.

Communication and Representation Area. First Cycle Block 9. Digital Literacy	
Evaluation Criteria	Objectives
EC 9.1. Interact virtually, becoming familiar with the use of different media and digital tools.	OBJ 1
EC 9.2. Interpret messages transmitted in digital format wondering about the intentionality of the sender.	OBJ 2
EC 9.3. Intuitively use different intuitive and visual digital tools or applications.	OBJ 3
Contents	
Approach to the use of digital applications and tools for different purposes: communication, learning and enjoyment; Healthy and responsible use of digital technologies; Reading and critical interpretation of images and information received through digital media; Exploration of the functions of technological devices and elements in their environment.	
Communication and Representation Area. Second Cycle Block 9. Digital Literacy	
Evaluation Criteria	Objectives
EC 9.1. Interact virtually, becoming familiar with the use of different media and digital tools.	OBJ 1
EC 9.2. Interpret messages transmitted by means of artistic representations or manifestations, or in digital format, recognizing the intentionality of the sender and showing a curious and responsible attitude.	OBJ 2
EC 9.3. Express themselves creatively using different intuitive and visual digital tools or applications to express themselves creatively.	OBJ 3
EC 9.4. Know and apply the basic rules for the use of technological tools:times of use, contexts and moments of use, type of application and suitable contents.	OBJ 2
Contents	
Approach to the use of digital applications and tools for the following different purposes: creation, communication, learning and enjoyment; Healthy and responsible use of digital technologies; Approach to the use of digital tools in creative processes; Critical reading and interpretation of images and information received through digital media; Educational function of technological devices and elements in their environment.	

**Table 4:** Assessment criteria and contents in the Growth in Harmony Area during the first cycle.

Growth in Harmony Area. First Cycle Block 3. Healthy Lifestyle Habits for Self-Care and Environmental Protection	
Evaluation Criteria	Objectives
EC 3.1. Practice a healthy lifestyle and promote comprehensive well-being through a balanced and enjoyable diet, adequate and independent rest, a love of cleanliness and appropriate attire.	OBJ 3
EC 3.3. Demonstrate awareness in identifying one’s own needs, progressing in autonomy, security, and intentionality in meeting them.	OBJ 3
EC 3.5. Acquire habits related to cleanliness, care, and order in one’s environment.	OBJ 3
Contents	
Adoption of sustainable and eco-socially responsible habits related to nutrition, hygiene, personal grooming, rest and cleanliness of space; Actions that promote health and generate well-being. Interest in presenting a healthy and tidy appearance; Identification and recognition of personal characteristics and qualities for acceptance of one’s own image, positively valuing differences; Progressive acquisition of autonomy in rest, eating, hygiene and cleanliness habits; Initiation into the practice of actions that promote interaction and the acquisition of healthy habits, such as body and environmental hygiene, proper nutrition, responsible consumption and the regulation of sleep and wakefulness, activity and rest and action and relaxation; Progressive adaptation of one’s own biological rhythms to group routines; Acquisition of routines related to commitment and autonomy, as follows: anticipation of actions and norms of social behavior during meals, rest, hygiene and travel; Identification of common risks in your environment and use of appropriate strategies and resources to avoid them.	
Block 4. Socio-Emotional Interaction in the Environment. Life with Other People	
Evaluation Criteria	Objectives
EC 4.3. Establish healthy bonds and attachment relationships, demonstrating caring and empathetic attitudes toward others.	OBJ 4
Contents	
Early emotional bonds. Openness and interest in others. Feelings of belonging and emotional connection with role models; Interest and willingness to establish respectful, caring and mutual relationships with others.	

**Table 5:** Assessment criteria and content in the Growth in Harmony Area during the second cycle.

Growth in Harmony Area. Second Cycle Block 3. Healthy Lifestyle Habits for Self-Care and Environmental Protection	
Evaluation Criteria	Objectives
EC 3.2. Recognize health habits, healthy eating, personal hygiene and well-being, using spaces and materials appropriately.	OBJ 3
EC 3.3. Identify risk situations and respond consistently to them, accepting the rules.	OBJ 3
EC 3.4. Perform activities related to self-care and environmental care with a respectful attitude, showing self-confidence and initiative.	OBJ 3
EC 3.5. Respect the time sequence associated with daily events and activities, adapting to established group routines and developing respectful behaviors toward others.	OBJ 3
EC 3.6. Perform daily activities with initiative and autonomy, collaborate on tasks, and accept the rules.	OBJ 3
Contents	
Recognition of the body’s basic needs, as follows: hygiene, clothing, food, rest, and activity; Acceptance of established standards of behavior during meals, travel, rest and hygiene; Appreciation of the need to develop in healthy spaces, identifying the conditions that characterize them. Collaboration in maintaining clean and orderly environments; Sustainable and eco-socially responsible habits and practices related to food, hy-giene, physical activity, rest, self-care and environmental care; Identification of dangerous situations and adoption of appropriate behaviors to prevent accidents and illnesses; Responsible and appropriate use of instruments, tools and facilities to prevent accidents and avoid risky situations; Structured physical activity with varying degrees of intensity; Routines: sequential planning of actions to complete a task; standards of social behavior regarding eating, rest, hygiene, travel, etc.	

## Data Availability

The data and original contributions presented in this study are included in the article. Further inquiries can be directed to the corresponding author.

## Abbreviations

The following abbreviations are used in this manuscript:

HE	Health Education
ECE	Early Childhood Education
PE	Physical Education
CLC	Competence in Linguistic Communication
MC	Multilingual Competence
DC	Digital Competence
PSLLC	Personal, Social and Learning to Learn Competence
CC	Citizenship Competence
EC	Entrepreneurial Competence
CCAE	Competence in Cultural Awareness and Expression
MCCSTE	Mathematical Competence and Competence in Science, Technology and Engineering

## References

[1] Wold Health Organization (WHO) (1998). Health Promotion. Glossary.

[2] Rieck G., Lundin J. (2025). Health Education.

[3] Miranda López F., Monroy Magaldi D. (2024). La Educación Para la Salud y el Bienestar en las Escuelas Primarias. Experiencias y Percepciones Desde las Comunidades Educativas.

[4] Ley Orgánica 3/2020 (2020). Por la Que se Modifica la Ley Orgánica 2/2006, de 3 de Mayo, de Educación.

[5] (2020). Por el que se Establecen la Ordenación y el Currículo de la Educación Infantil en la Comunidad Autónoma de Galicia.

[6] Alonso A., Martínez S., Pérez J. (2022). Educación nutricional en la infancia: Estrategias y resultados de intervención. Rev. De Salud Pública.

[7] Llorente M., Gómez J., Sánchez R. (2021). La salud emocional en la educación infantil: Un enfoque preventivo. J. Child Dev..

[8] Moya A., Ruiz M., García L. (2023). La educación para la salud en el contexto escolar infantil: Metodologías y prácticas efectivas. Educ. Salud.

[9] Rodríguez A., Pérez I., Jiménez P. (2021). El papel de la familia en la educación para la salud en la infancia. Psicol. Educ..

[10] (2020). Por el Que se Establece la Ordenación y las Enseñanzas Mínimas de la Educación Infantil.

[11] European Commission (2018). Communication on Promoting Healthy Lifestyles in the European Union.

[12] European Centre for Disease Prevention and Control (2020). Hand Hygiene in Early Childhood Settings.

[13] European Food Safety Authority (2021). Safe Food and Sustainable Food Systems Through Transparent, Independent and Trustworthy Scientific Advice.

[14] European Agency for Safety and Health at Work (2019). Healthy Workplaces for All Ages.

[15] Department for Education (2017). Early Years Foundation Stage (EYFS) Framework.

[16] Llorent V. (2013). La educación infantil en Alemania, España, Francia e Inglaterra. Estudio comparado. Rev. Española De Educ. Comp..

[17] Ministerio Federal de Educación e Investigación de Alemania (2019). Currículo Para la Educación Infantil.

[18] Ministère de l’Éducation Nationale de Francia (2020). Programas de Educación en la École Maternelle.

[19] Gavidia V. (2016). Los ocho Ámbitos de la Educación para la Salud en la Escuela.

[20] Rubio S., Mora M. (2019). Educación Para la Salud y el Consumo en Educación Infantil.

[21] Ministerio de Sanidad (2023). Guía de Escuelas Promotoras de Salud.

[22] Arufe Giráldez V. (2020). ¿Cómo debe ser el trabajo de Educación Física en Educación Infantil?. Retos.

[23] López-Sobaler A.M., Aparicio A., Salas-González M.D., Kohen V.L., Bermejo López L.M. (2021). Obesidad en la población infantil en España y factores asociados. Nutr. Hosp..

[24] Tejero González J.M. (2021). Técnicas de Investigación Cualitativa en los Ámbitos Sanitario y Sociosanitario.

[25] Tree Bressen K. (2006). Group Facilitation Primer.

[26] Reyes-López O., Hernández-Moncada M.C. (2021). Formato. Validación de Contenido por Juicio de Expertos. Instrumentos Cualitativos.

[27] Organización Mundial de la Salud [OMS] (2022). Directrices de la OMS Sobre los Servicios de Salud Escolar.

[28] García M., López A. (2022). Formación del profesorado en educación física en la etapa de educación infantil en España: Desafíos y perspectivas. Rev. Educ. Física Deporte.

[29] Fernández J., Sánchez R. (2023). Barreras en la formación del profesorado de educación física en educación infantil en España. Rev. Iberoam. Educ..

[30] Universidad de Santiago de Compostela (2025). Estudios del Grado en Maestro/a de Educación Infantil.

[31] Alonso V., Pérez M. (2022). La formación del profesorado en educación física en la educación infantil: Una revisión de la literatura. Rev. Investig. Educ..

[32] López C., Fernández E. (2023). Percepciones del profesorado sobre su formación en educación física en la etapa infantil en España. Rev. Estud. Pedagógicos.

[33] Pérez L., Gómez D. (2023). La importancia de la formación continua en educación física para docentes de infantil en España. Rev. Innovación Educ..

[34] Martínez P., Ruiz S. (2022). La preparación docente en educación física para la educación infantil en Europa: Un análisis comparativo. Eur. J. Phys. Educ..

[35] Kovačević T., Müller H. (2022). Teacher training gaps in early childhood physical education across Europe. Eur. J. Teach. Educ..

[36] Schmidt A., Weber M. (2022). Formación inicial y continua del profesorado en educación física en Europa: Análisis de políticas y prácticas. Eur. J. Educ..

[37] Svensson T., Johansson K. (2023). Enhancing physical activity in preschool settings across Scandinavia: Strategies and outcomes. Nord. J. Child. Educ..

[38] Pons R., Arufe V. (2016). Análisis descriptivo de las sesiones e instalaciones de psicomotricidad en el aula de educación infantil. Sport. Sci. Tech. J. Sch. Sport Phys. Educ. Psychomot..

[39] Fernández A., Ruiz L. (2024). Impacto de las horas adicionales de actividad física en el desarrollo infantil en centros educativos de Madrid. Rev. Psicol. Educ..

[40] García M., López P. (2023). La importancia de incrementar las horas de actividad física en la educación infantil en España. Rev. Española Educ. Física.

[41] López R., Martínez D. (2025). Evaluación de programas de incremento de horas de actividad física en centros de educación infantil en Barcelona. Rev. Int. Educ..

[42] Pérez J., Gómez S. (2024). La influencia de las horas de actividad física en el bienestar y desarrollo de niños en centros educativos de Valencia. Rev. Salud Infant..

[43] Organización Mundial de la Salud [OMS] (2024). Health-Enhancing Physical Activity in the European Union.

[44] European Commission (2024). Policy Recommendations for Increasing Physical Activity in Early Childhood Education.

[45] Müller S., Schmidt J. (2022). Promoting physical activity in early childhood education: A European perspective. Eur. J. Phys. Educ..

[46] Estudio ALADINO (2023). Estudio Sobre la Alimentación, Actividad Física, Desarrollo Infantil y Obesidad en España 2023.

[47] Universidad de Zaragoza (2024). España Encabeza el Ranking Europeo de Obesidad Infantil.

[48] Díaz-Vicario A., Gairín Sallán J. (2021). La formación en salud y seguridad del profesorado para la gestión de centros educativos seguros y saludables. Innovación Educ..

[49] Soriano Sánchez J.G. (2025). La Educación Para la Salud en el Currículo de Educación Infantil.

[50] Organización de las Naciones Unidas (2025). Objetivos de Desarrollo Sostenible.

[51] Organización Mundial de la Salud [OMS] (2006). Skills for Health: Skills-Based Health Education Including Life Skills.

[52] UNESCO (2021). Global Standards for Health-Promoting Schools. Joint Resource Package.

[53] Rowling L., Samdal O. (2021). Filling the black box of implementation for health-promoting schools. Health Educ..

[54] St. Leger L. (2010). Reducing the barriers to the expansion of health-promoting schools by focusing on teachers. Health Educ..

[55] Drouka A., Brikou D., Causeret C., Al Ali Al Malla N., Sibalo S., Ávila C., Yannakoulia M. (2023). Effectiveness of school-based interventions in Europe for promoting healthy lifestyle behaviors in children. Children.

[56] Zurc J., Laaksonen C. (2023). Effectiveness of health promotion interventions in primary schools—A mixed methods literature review. Healthcare.

[57] Paakkari L., George S. (2022). Health literacy in schools: The importance of teacher competence. Health Educ..

[58] Grauduszus M., Koch L., Wessely S., Joisten C. (2024). School-based promotion of physical literacy: A scoping review. Front. Public Health.

[59] Gunawardena H., Voukelatos A., Nair S., Cross S., Hickie I.B. (2023). Efficacy and effectiveness of universal school-based wellbeing interventions in Australia: A systematic review. Int. J. Environ. Res. Public Health.

